# Age-Related Effect of Viral-Induced Wheezing in Severe Prematurity

**DOI:** 10.3390/children3040019

**Published:** 2016-10-20

**Authors:** Geovanny F. Perez, Amisha Jain, Bassem Kurdi, Rosemary Megalaa, Krishna Pancham, Shehlanoor Huseni, Natalia Isaza, Carlos E. Rodriguez-Martinez, Mary C. Rose, Dinesh Pillai, Gustavo Nino

**Affiliations:** 1 Division of Pulmonary and Sleep Medicine, Children’s National Medical Center, Washington, DC 20010, USA; ajain@childrensnational.org (A.J.); Rmegalaa@childrensnational.org (R.M.); shuseni@chlildrensnational.org (S.H.); mrose@childrensnational.org (M.C.R.); dpillai@childrensnational.org (D.P.); gnino@childrensnational.org (G.N.); 2 Department of Pediatrics, George Washington University School of Medicine and Health Sciences, Washington, DC 20010, USA; bkurdi@childrensnational.org (B.S.); 3 Department of Integrative Systems Biology and Center for Genetic Medicine Research, George Washington University, Washington, DC 20010, USA; 4 Center for Genetic Research Medicine, Children’s National Medical Center, Washington, DC 20010, USA; 5 Division of Pediatric Pulmonology, University of Kentucky, Lexington, KY 40536, USA; Pancham@zoho.com; 6 Division of Neonatology, Children’s National Medical Center, Washington, DC 20010, USA; nisaza@childrensnational.org; 7 Department of Pediatrics, School of Medicine, Universidad Nacional de Colombia, Bogota 111321, Colombia; carlos2671@gmail.com; 8 Department of Pediatric Pulmonology and Pediatric Critical Care Medicine, School of Medicine, Universidad El Bosque, Bogota 111321, Colombia; 9 Research Unit, Military Hospital of Colombia, Bogota 111321, Colombia

**Keywords:** prematurity, rhinovirus, respiratory syncytial virus

## Abstract

Premature children are prone to severe viral respiratory infections in early life, but the age at which susceptibility peaks and disappears for each pathogen is unclear. **Methods:** A retrospective analysis was performed of the age distribution and clinical features of acute viral respiratory infections in full-term and premature children, aged zero to seven years. **Results:** The study comprised of a total of 630 hospitalizations (n = 580 children). Sixty-seven percent of these hospitalizations occurred in children born full-term (>37 weeks), 12% in preterm (32–37 weeks) and 21% in severely premature children (<32 weeks). The most common viruses identified were rhinovirus (RV; 60%) and respiratory syncytial virus (RSV; 17%). Age-distribution analysis of each virus identified that severely premature children had a higher relative frequency of RV and RSV in their first three years, relative to preterm or full-term children. Additionally, the probability of RV- or RSV-induced wheezing was higher overall in severely premature children less than three years old. **Conclusions:** Our results indicate that the vulnerability to viral infections in children born severely premature is more specific for RV and RSV and persists during the first three years of age. Further studies are needed to elucidate the age-dependent molecular mechanisms that underlie why premature infants develop RV- and RSV-induced wheezing in early life.

## 1. Introduction

Early-life viral respiratory infection is a potentially modifiable risk factor for the development of lower airway obstruction in infants and children, and it represents a major cause of morbidity and mortality within this age group [1]. Of special interest are young children born prematurely, as they are highly susceptible to respiratory viruses, particularly respiratory syncytial virus (RSV) and rhinovirus (RV), the most common pathogens causing acute respiratory illnesses and hospitalizations in this population [2,3,4,5]. Prior studies have shown viral respiratory infections in premature children are an increasing source of public health care utilization, due to multiple outpatient/emergency room visits and prolonged hospitalizations [2]. Notably, it is unclear until what age premature children remain highly vulnerable to respiratory viruses. Establishing the age range during which the predisposition to severe RV and RSV infections is still present in premature children can greatly benefit the design of preventive interventions and reduce the burden of severe viral respiratory illnesses in the 15 million babies born prematurely in the world each year [6,7].

Multiple studies have demonstrated that young age is a critical host factor in determining severity and clinical presentation of RSV and RV in children [8]. Indeed, RSV is the most frequent cause of lower respiratory tract infections (LRTIs) in young infants, and RV is the most commonly identified virus involved in wheezing exacerbations in older infants, preschool-aged and school-aged children [8]. Longitudinal studies have also established that the age at which the viral infection occurs influences the probability of experiencing wheezing and asthma later in life [9,10,11]. Interestingly, prior studies have not specifically focused on investigating the role of young age in the severity and clinical presentation of RSV and RV in premature children. The goal of the present study is to define the age distribution at which most viral hospitalizations occur in premature children. We speculated that children born prematurely have a distinctive distribution and clinical presentation relative to children born at term.

## 2. Materials and Methods

We conducted a retrospective, cross-sectional analysis of a cohort of children less than seven years of age, admitted with viral respiratory infection, at Children’s National Medical Center (CNMC) during 2014. At the discretion of a clinician, viral PCR were performed on subjects who presented to the hospital with suspected viral respiratory tract infection. We only included children with positive PCR for any of the viruses in our panel including RV, RSV, human metapneumovirus (HMPV), influenza A/B, parainfluenza 1–3, and adenovirus. Patients with significant co-morbidities, such as cardiorespiratory conditions (other than prematurity), genetic syndromes or immunosuppression, were excluded from the study. This study was approved by the Institutional Review Board at CNMC. The primary goal of the study was to determine the age range at which most of the viral-induced hospitalizations occur in premature children. Secondary analysis investigated the effect of young age on the probability to develop viral induced (RV or RSV) wheezing in severely premature children.

### 2.1. Clinical and Demographic Variables

Clinical and demographic variables were obtained by reviewing electronic medical records (EMR) at CNMC. Demographic variables comprised of gestational age (GA) in weeks, age, gender, and ethnicity. For the purpose of the study, clinical parameters were characterized as binary outcomes for the presence of wheezing, and the classification of severe prematurity defined a priori by a GA of less than 32 weeks, to include extremely preterm and very preterm subjects, based on the World Health Organization (WHO) definition of prematurity [12,13].

### 2.2. Statistical Analysis

Data were analyzed using the software SAS (Version 9.3; SAS Institute Inc., Cary, NC, USA). Descriptive statistics were used to calculate the prevalence of each virus. Collected demographic and clinical data were compared with the use of a Chi-square test (categorical variables), *T*-test, or Wilcoxon rank-sum test, as appropriate for continuous variables. Multivariate models (logistic regression) were built to examine the link between prematurity and viral-induced wheezing, adjusted for pertinent co-variates. Significance was taken at the *p* < 0.05 level.

## 3. Results

### 3.1. Epidemiology of Viral Respiratory Infections According to Gestational Age (GA)

**Figure 1:**
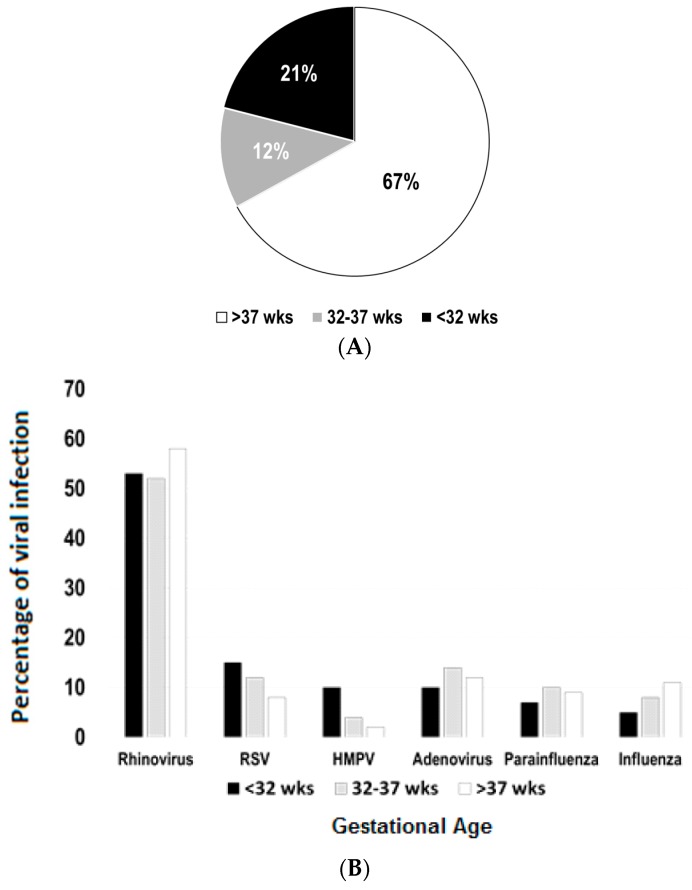
Epidemiology of viral respiratory infections according to gestational age (GA). (**A**) Sixty-seven percent of all viral-induced hospitalizations (n = 630) occurred in children born full-term (>37 weeks), 12% in preterm (32–37 weeks) and 21% in severely premature children (<32 weeks); (**B**) distribution of viral pathogens according to GA, presented as percentages of each virus (excluding cases of mixed infections; n = 69); RSV = respiratory syncytial virus; HMPV = human metapneumovirus.

The study comprised of a total of 630 hospitalizations in children aged seven years or less (n = 580 children). As shown in Figure 1A, our initial results demonstrated that 67% of these hospitalizations (421/630) occurred in children born full-term, 12% in preterm (76/630) and 21% in severely premature children (132/630). A stratified analysis of viral pathogen distribution was conducted according to GA group (severely premature, pre-term and full-term). After excluding cases of mixed viral infections (n = 69), we identified RV as the predominant pathogen causing viral-induced hospitalization in all GA groups. RV was identified in 53%, 52% and 58% of the cases in severely premature, pre-term and full-term children, respectively (Figure 1B). RSV and HMPV appeared to be more common in children born severely premature (RSV 12% and HMPV 10%) than in full-term individuals (RSV 8% and HMPV 2%). Other viruses (adenoviruses, parainfluenza 1–3 and influenza A/B) did not have a marked difference in distribution according to GA groups (Figure 1B).

### 3.2. Age Distribution Analysis of Viral Respiratory Infections According to GA

**Figure 2:**
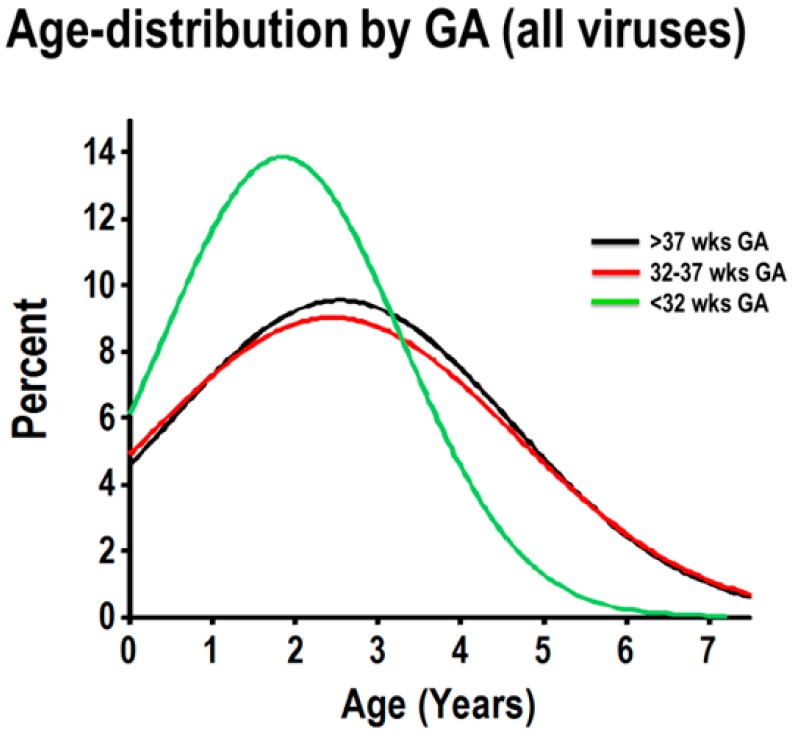
Normalized age distribution analysis of viral respiratory infections according to GA. Combined frequency of viral respiratory infections in children born full-term (black), preterm (red) and severely premature children (green).

To examine the age distribution of hospitalizations due to respiratory viruses according to GA, we first combined all cases of PCR-confirmed viral respiratory infection (all viruses) to model the age distribution according to GA groups (severely premature, pre-term and full-term), using normalized histograms. As shown in Figure 2, children born full-term and preterm had the same age frequency distribution. Within these two GA groups, viral-induced hospitalizations occurred throughout all included ages, with about 50% of the cases being present in children zero to three years old, and the remaining cases occurring in older individuals (Figure 2). In contrast, viral-induced hospitalizations in children born severely premature had a strikingly different age distribution, with a distinct peak frequency at a younger age (≈50% of cases occurred between 0 and 1.8 years of age) and the majority of cases (≈80%) occurring in children three years of age or less (Figure 2).

### 3.3. Age-Related Effect of Viral-Induced Wheezing in Severe Prematurity

**Figure 3:**
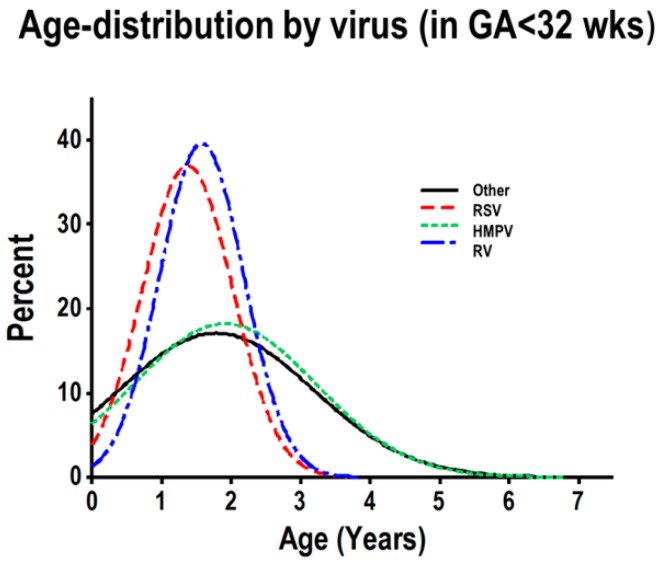
Normalized histogram of age distribution analysis of viral respiratory infections, according to virus, in severely premature children. Individual rhinovirus (RV; blue), RSV (red), HMPV (green) and other viruses (black).

To further examine the distinct age distribution observed in severely premature children, hospitalized due to viral respiratory infections, we modeled the age distribution according to viral pathogens (RV, RSV, HMPV and other viruses) in severely premature children. These analyses identified that RV and RSV had very similar age distributions in children born severely premature (Figure 3), with a pronounced frequency peak in the first three years of life. The vast majority of RV-/RSV-induced hospitalizations (>80%) occurred in children three years old or less (Figure 3). The normalized age frequency distribution of HMPV and other viruses (adenoviruses, parainfluenza 1–3 and influenza A/B) did not exhibit a peak in the first three years of life (Figure 3). None of the viruses showed a peak in the first three years of life in children born preterm or full-term (data not shown).

**Figure 4:**
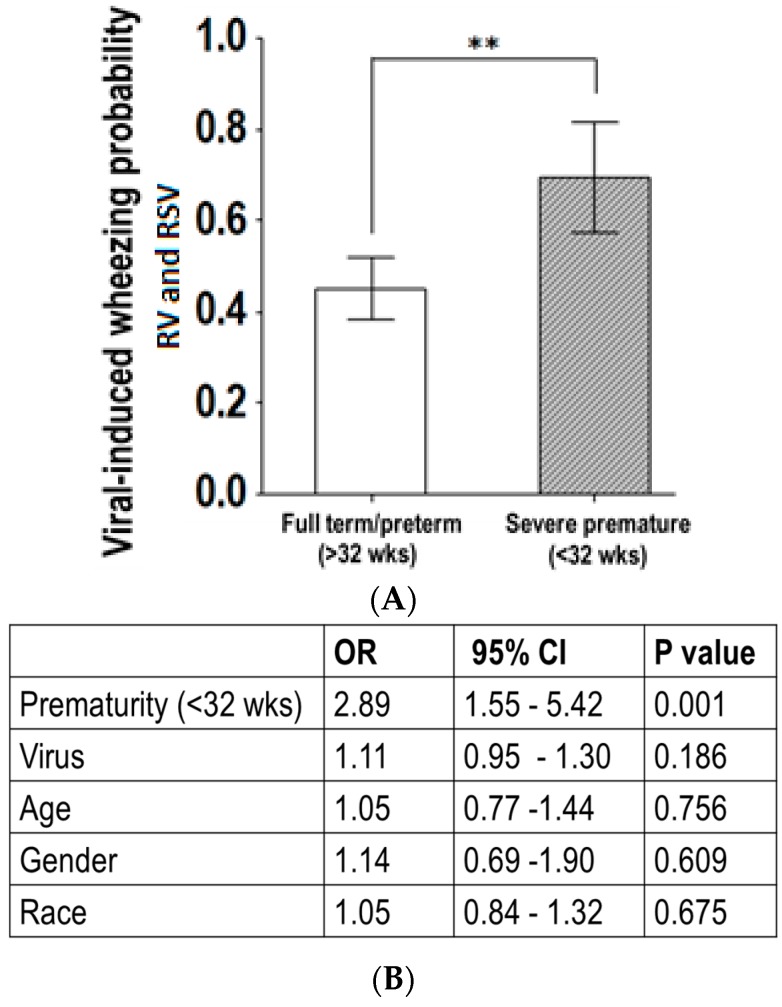
Age-related effect of viral-induced wheezing in severe prematurity. (**A**) Bars (95% CI of the mean) show the probability of virus-induced wheezing was overall higher in severely premature children less than three years old; ** *p* < 0.01; (**B**) Multivariate logistic regression identified the link between RV- or RSV-induced wheezing and severe prematurity was independent of gender, race and viral pathogen identified.

We then conducted a subanalysis of the data, from the group of severely premature children with RSV and RV infections. Figure 4 illustrates that RV- or RSV-induced wheezing, along with sub-costal retractions, were more likely to be present in severely premature children three years old or less, compared to pre-term or full-term children. Additionally, multivariate logistic regression identified that the link between RSV/RV infection and wheezing in severely premature children aged three years or less was independent of gender and race (adjusted OR = 2.89; *p* < 0.05).

## 4. Discussion

Although numerous studies have demonstrated that premature birth predisposes a child to viral infections in early life [2,3,4,5], none of these studies have examined at which age premature children are susceptible to respiratory viruses. Studying a population of hospitalized children with PCR-confirmed respiratory viral infections (n = 580 children), we identified that individuals born severely premature (<32 weeks GA) have a higher percentage of RSV and RV infections in their first three years of life, relative to pre-term and full-term children. Specifically, modeling the frequency of each virus by age, we identified that the age distribution of RV and RSV was strikingly similar in children born severely premature (Figure 2 and Figure 3). In fact, >80% of the RV-/RSV-induced hospitalizations in severely premature children occurred in individuals aged three years or less. Moreover, severely premature children aged three years or less infected with RV or RSV were significantly more likely to have wheezing than age-matched pre-term or full-term children (Figure 4). These age-related effects were not seen with any of the other respiratory viruses examined, including HMPV, influenza A/B, parainfluenza 1–3 or adenovirus. Collectively, our data provides new evidence that individuals born prematurely are specifically more susceptible to RV and RSV than to other respiratory viruses. The susceptibility to RV/RSV infections in prematurity is greatly related to early development, since it is mostly present during the first three years of life.

Our results reflect the current body of knowledge on the reasons why viral-induced infections could be associated with GA. For instance, prematurity is a known risk factor for viral LRTIs. Prior studies suggest this may be due to airway immune cytokine dysregulation. In a study by Perez et al., it was observed that premature children have dysregulated T helper (Th) 2 and Th17 airway cytokine immune responses to RV infection, which might be a potential reason for increased sensitivity to RV in premature children during early life [13]. Furthermore, systemic innate immune responses [14,15] and antibody-mediated immunity [16], which depends on maternal transfer of specific IgG across the placenta, seem to be dysregulated in premature babies [14,15,16]. Prior studies have shown cytokine responses in early life are largely driven towards a Th2 phenotype, leading to lower production of Th1 antiviral cytokines such as IFNɣ [17,18,19]. This deficient Th1 cytokine production in neonates may explain the susceptibility of this age group to certain pathogens such as RSV and HMPV [20,21]. These findings are in agreement with previous reports of an unbalanced nasal airway Th1/Th2 response during RSV and RV infection in young children [22,23]. The effect of RV and RSV inducing Th2 airway immune responses has also been reported in animal models [24,25], including studies investigating viral-induced airway hyper-reactivity in neonatal mice [25]. RV-/RSV-induced Th2 responses may facilitate the development of airway hyper-reactivity, due to the secretion of airway epithelial cytokines (e.g., IL-25, TSLP) and classical Th2 cytokines (IL-13), leading to increased mucus secretion and bronchoconstriction [25], which are clinically manifested as viral-induced wheezing in young children.

In addition to dysregulated immune responses, other age-related factors that appear to be important in determining the likelihood of wheezing are decreased pulmonary reserve and low lung volumes [26,27,28,29]. Premature birth is associated with alveolar simplification, due to impaired secondary septation, leading to reduced lung capacity and increased airway resistance, owing to altered alveolar airway radial traction [29]. These findings have been confirmed through the use of pulmonary function testing [26,27,28,29] and studies that have examined the effects of viral infections have shown airway resistance is significantly increased during respiratory infections in premature infants [29]. In addition, studies primarily based on the report of wheezing have identified that RV and RSV are associated with frequent health care utilization, mostly due to recurrent lower airway respiratory symptoms, particularly in young children born prematurely [2]. In this regard, it is noteworthy that Drysdale et al. showed infants born prematurely with a history of RV or RSV LRTI underwent an overall increase in healthcare utilization during infancy, and had significantly greater health-related costs of care [8]. These increased health costs were largely attributed to a greater number of medical visits and persistent airway reactivity (wheezing) at follow-up [2].

Although there is a paucity of data investigating the link between age and prematurity in the context of viral-induced wheezing, there is still compelling evidence demonstrating the maturational effects of viral-induced wheezing in children [1,8,9,10,11]. The Tucson cohort, which is the largest asthma birth cohort in the USA [9], has identified three years of age as a critical age period to define childhood asthma phenotypes [9]. Specifically, longitudinal studies from this asthma cohort described four main phenotypes based on the occurrence of wheezing and lower respiratory illnesses before age three, and active wheezing at age six. The four wheezing phenotypes included: (1) no wheeze from birth to age six (never wheeze); (2) wheezing before age three only (transient early wheeze); (3) wheeze at age six only (late-onset wheeze); and (4) wheezing before age three and wheezing at age six (persistent wheeze). The risk of subsequent asthma was greatly influenced by these phenotypes, and the greatest incidence is seen in those children with persistent wheezing [9]. Another large longitudinal cohort, the Childhood Origins of Asthma (COAST) study, also identified the first three years of life as a critical period in which viruses (particularly RV and RSV) can have a great influence in the risk of experiencing wheezing beyond childhood [10,11]. In fact, this study observed that during the first three years of life, children with RV-induced wheezing illnesses had a 10-times-higher risk of developing asthma later in life [11]. Our study is in overall agreement with these studies, since we observed that susceptibility to develop wheezing during RV/RSV in prematurity was mostly present during the first three years of life, indicating this time window is critical in determining the airway responses to these viruses and therefore could also be a likely future asthma risk.

Our study has a number of strengths and some limitations. We included a relatively large cohort of children hospitalized due to viral respiratory infections, comprised of a significant number of prematurely born individuals. We were able to investigate clinical manifestations (e.g., wheezing) and correlate this with viral-specific pathogens (determined by PCR) and GA. The main limitation of the present study is the retrospective collection of clinical data. However, because the data were taken from EMR and the key variables analyzed are hard variables (i.e., age, GA, viral PCR-result), it is unlikely that the retrospective collection significantly compromised the validity of the results due to misclassifications of disease status (i.e., prematurity or RV or RSV infected). It is important to mention the fact that the study was conducted in a specialized, tertiary referral hospital, and therefore, the patients included represent the extreme of the spectrum of severity of all patients with viral respiratory infection, which could potentially limit the generalizability of the results. However, since the results of our study are similar to the results of previous studies linking prematurity with RV and RSV [2,3,4,5], we believe that extrapolation to other contexts is possible. Due to the nature of the cross-sectional design of the study, we were unable to define at which age GA stops being the defining factor for frequent virus-induced wheezing. Future longitudinal studies are needed to elucidate this important question.

In summary, our results indicate children born severely premature (<32 weeks GA) have a vulnerability to RV and RSV that persists during the first three years of life. Further studies are needed to elucidate the exact molecular mechanisms that underlie why severely premature babies develop wheezing and respiratory distress during viral respiratory infections in early childhood.
